# Evaluating a capacity building program on women’s health for displaced community health workers in fragile settings in Lebanon

**DOI:** 10.1186/s12960-021-00585-0

**Published:** 2021-03-21

**Authors:** Hady Naal, Rebecca Mendelsohn, Dayana Brome, Aya Noubani, Dana Nabulsi, Dina Muhieddine, Shadi Saleh

**Affiliations:** 1 Global Health Institute, American University of Beirut, Beirut, Lebanon; 2 Gerald R. Ford School of Public Policy, University of Michigan, Ann Arbor, USA; 3 Faculty of Health Sciences, American University of Beirut, Beirut, Lebanon

**Keywords:** Task-shifting, Capacity building, Program evaluation, Syrian refugees, Community members, Lebanon

## Abstract

**Background:**

Displaced populations in fragile settings experience health disparities that are seldom attended to. Task-shifting, which involves training non-specialized community health workers (CHW) to deliver basic education and health services is a favorable strategy to address this problem, however very little data exist on this topic in the Middle East region. We conducted a long-term evaluation of the Women’s Health Certificate delivered to Syrian refugees and host community in informal tented settlements in Lebanon under the Mobile University for Health (MUH) program. The training was delivered through a mobile classroom approach that incorporated a blended learning modality.

**Methods:**

We collected short-term data from the 42 trained CHW (knowledge assessments and satisfaction measures) during the delivery of the intervention between March and August 2019, and long-term data (semi-structured interviews with 8 CHW and focus group discussion with 9 randomly selected community members) one year later between July and August 2020. The evaluation approach was informed by the Kirkpatrick evaluation model, and the qualitative data were analyzed using qualitative content analysis.

**Results:**

Data from the CHWs and community members were triangulated, and they showed that the training enhanced access to education due to its mobile nature and provided opportunities for CHWs to engage and interact with learning material that enhanced their knowledge and favorable behaviors regarding women’s health. In turn, CHWs were empowered to play an active role in their communities to transfer the knowledge they gained through educating community members and providing women’s health services and referrals. Community members benefited from the CHWs and called for the implementation of more similar training programs.

**Conclusion:**

This is one of few studies reporting a long-term community-level evaluation of a task-shifting program on women’s health among displaced populations in Lebanon. Our findings support the need to increase funding for similar programs, and to focus on delivering programs for a variety of health challenges. It is also essential to enhance the reach and length of recruitment to wider communities, to design concise, interactive, and engaging sessions, and to provide tools to facilitate circulation of learning material, and resources for referrals to health services.

## Introduction

The global health challenges associated with widespread displacement are a major concern for health practitioners, policy-makers, researchers, and civil societies around the world [1]. Since 2011, the civil war in Syria has furthered these concerns, and as the conflict approaches its tenth year, the catastrophic humanitarian effects of nearly a decade of violence are stronger than ever [2]. Lives have been lost, livelihoods and infrastructures have been destroyed, and healthcare systems have been unduly burdened. While the consequences of the war have been felt around the world, as thousands of displaced Syrians have sought refuge in the European Union, United States, and Canada, Syria and its neighboring countries undoubtedly endure the most significant burdens on multiple levels [3].

So far, roughly 12 million Syrians have been displaced, including approximately 6.5 million internally displaced within Syria, and 5.5 million that have sought refuge in countries throughout the Middle East and North Africa (MENA) region [4]. Most significantly, Lebanon, which is the focus of this study, and which has been affected by several conflicts in the MENA region, now hosts over 1.5 million Syrian refugees, amounting to approximately 25% of its population [5]. Such high levels of displacement, along with subsequent settling of populations in specific areas, have led to large-scale problems such as overcrowding, poor sanitation, limited access to food and basic necessities, and an overstretched healthcare system, to name a few. In turn, and as a result of the harsh and protracted nature of the war in Syria, many Syrian refugees experience increasingly complex and diverse health disparities [4, 6, 7]. According to a recent systematic review assessing the health needs of Syrian refugees in the MENA region, women's health has been identified as one of the most prevalent and pressing health concerns [4]. The study suggested that a lack of available women’s health services, including limited access to specialists, has increased the salience of related health problems. In this regard, and given the increased insecurity and long-term disruptions to healthcare access, women and girls in humanitarian settings face a unique set of health challenges. For example, those related to general health maintenance, reproductive health, and pregnancy to name a few [8], are exacerbated by lack of access to healthcare services and education due to the high cost of care, geographical limitations, lack of knowledge about how to access services, and restricted movement [4].

In addition to the challenges refugees themselves experience in trying to access healthcare services, several other factors have limited the ability of the healthcare system in Lebanon to respond to their needs. For example, Lebanon’s healthcare system is mostly privatized and is largely based on out-of-pocket expenditures [8]. Even before Lebanon became host to the largest concentration of refugees in the world, its healthcare system struggled to provide universally accessible and comprehensive health services [8]. As a result, costs remain high, and a sustained lack of trained staff, medication, and equipment, have made accessing high quality services difficult [9]. Although the UNHCR does cover essential health services to Syrian refugees through active primary healthcare centers, many refugees are unable to access these services due to geographical barriers, lack of awareness, and lack of health education to name a few [10]. Given these challenges, there is a need to strengthen the capacity of health workers within their communities to be able to provide non-specialized services as a way to overcome the barriers to accessing healthcare services.

Task-shifting, which involves the delegation of health promotion and health services from licensed medical professionals to trained yet non-specialized Community Health Workers (CHW), has emerged as an effective strategy to mitigate public health challenges that arise due to workforce shortages and other access issues [11, 12]. Studies suggest that training CHWs may help increase service coverage, health-related education, and knowledge sharing among at-risk communities [11, 13]. Positive impacts of such initiatives have been reported in the literature, such as successful reproductive and maternal health interventions within sub-Saharan African countries, including Tanzania, Mozambique, Zambia, and Ethiopia [11, 14]. While evidence suggests that global health capacity building initiatives are beneficial, little is known about their success in the MENA region and especially regarding women’s health in refugee populations, given the scarcity of case studies on this issue [13]. In fact, based on a recent systematic review, there is an increasing need for data on the effectiveness of capacity building programs that target CHWs, given their potential to provide non-specialized and essential services in low-resource settings [15].

Key studies which indicate that training CHW to deliver basic health services and to promote health may be an effective way to address gaps in the health workforce, have largely been conducted in sub-Saharan Africa and South Asia and among non-refugee populations [11, 16, 17]. The implementation and evaluation of CHW training programs in refugee settings in the MENA region therefore must be a priority to inform public health researchers and professionals, humanitarian actors, and policy-makers. CHW are often able to develop good relationships with members of their communities, and therefore are able to deliver tailored and context-specific health education and related basic services [13]. A more robust understanding of the impacts associated with these capacity building initiatives will enable more effective training design and delivery, better allocation of resources, and ultimately improved health outcomes for refugees in the MENA region and other settings with similar development landscapes [18].

In response to the aforementioned challenges, the Global Health Institute (GHI) at the American University of Beirut (AUB) designed, developed, and delivered the Mobile University for Health (MUH), a capacity building program under its Academy Division. Knowing that women in displaced communities and low-resource settings face barriers to accessing training and education due to care responsibilities and gendered power relations [19], MUH focuses on strengthening the health-related abilities of female Syrian refugees and host communities in Lebanon. MUH empowers women and communities through fostering an able health workforce among female Syrian refugees and host communities to compensate for their lost opportunities for higher education and prepares them to deliver basic education and services in specific health topics. The present study evaluates the long-term effectiveness and impact of the first implementation of this program, which centered around Women’s Health, on the individual and community levels.

## Methods

### Program development

The MUH program directly responds to the most pressing health needs of Syrian refugees and host community members in Lebanon and neighboring countries, in terms of delivery method and training content.

The development of the program underwent multiple phases and was informed by several sources of data by initiatives led by GHI and findings in the literature. A systematic review of the literature was conducted, which clarified that the priority health needs of Syrian refugees fall under Mental Health, Women’s Health, and Non-Communicable Diseases [4]. This was then followed by validation meetings in February 2018 and roundtable discussions in May 218 with key stakeholders in Lebanon, such as directors and representatives of the Ministry of Public Health, Primary Healthcare Centers (PHC), non-governmental organizations, and humanitarian agencies, which pointed towards similar results.

Upon reaching consensus between involved parties on which topic should be offered first depending on its urgency, a curriculum development committee was formed, which informed the development of the certificate’s content. It was agreed that Women’s Health should take precedence, and hence a corresponding expert committee of AUB faculty members participated in this process; content for the rest of the topics was developed later, and consequently these certificates are currently being delivered to the target populations. The outcome was the development of four courses by two subject matter experts as part of the Women’s Health Certificate (WHC):

#### Women’s health maintenance

This module illustrates the significance and importance of women’s health maintenance and the application of basic screening techniques. In this module students learn how to identify and suggest management recommendations for health issues affecting women.

#### Reproductive health

This module enables students to understand reproductive health and to describe its significance especially in vulnerable settings. In this module students learn how to address health issues such as sexually transmitted infections, reproductive tract infections, common cancers that affect women, and their options for contraception.

#### Pregnancy

In this module, students learn how to conduct a complete prenatal and obstetrical history from pregnant women, describe screening tests, and follow-up for positive pregnancy experience. Additionally, students learn how to define perinatal mental health disorders, and how to prepare new mothers for effective breastfeeding.

#### Health at midlife

In this module, students learn how to support women experiencing midlife changes and anxiety, and how to define medical problems related to menopause and osteoporosis. Additionally, students learn about common myths and misconceptions regarding menopause, about the risk factors for pelvic organ prolapse, and about management options for women with pelvic organ prolapse.

The decision to offer a certificate was set in agreement with the project funder. This is because certificates are the most basic and foundational proof of knowledge regarding a given topic, and which are recognized by local, regional, an international entities and organizations. In this regard, the program intended to train women who were Syrian refugees or who were Lebanese and came from disadvantaged backgrounds so that they may serve as CHW to other individuals in their communities. In addition to that, it was crucial for the program to be accessible and easy to reach. With this in mind, the program was designed to be *mobile,* with the classroom being transported to the target community using innovative methods that emphasize on the integration of technology when delivering the sessions.

### Project implementation, population, and setting

Prior to implementation, the certificate underwent a pilot-testing phase with a sample of participants from the target group in order (1) to gauge the logistics and feasibility of delivering the training; (2) assess the learners’ feedback, and (3) make recommendations for improvement to optimize the delivery of the training.

Following the pilot phase, the training was delivered between March and August 2019 to two areas in Lebanon (Akkar and Beirut). A total of four courses were delivered for each cohort. In each certificate, participants received 20 lecture hours (mixed between pre-recorded videos and in-person lectures), 6 practicum-based clinical observation hours, 4 case studies and interactive hours (with in-person teaching assistant). All courses and activities were delivered in Arabic, which is the native language of the target population.

Two different cohorts of women CHW recruited from local PHC participated in the training, and were selected on the basis of meeting the following inclusion criteria:

o Having official documentation of legal refugee status.
p Being between the ages of 21 and 45.
q Having a minimum of middle school-level education.
r Living in Beirut or in Akkar.

The first (Beirut) and second (Akkar) cohorts included *n* = 19 and *n* = 23 participants, respectively. Almost half of the Beirut sample (52.6%) had a university degree, with the rest having received a minimum of grade 9-level education. The mean age of participants was 31 with a standard deviation of 5.0. As for the Akkar sample, a smaller number held a university degree (30.4%), with the rest having received a minimum of grade 9-level education. The mean age of participants was 34.2 with a standard deviation of 9.8. No dropouts were reported from the training program and attendance was stable, since almost all participants attended all of the sessions.

### Data collection tools and procedures

We used a mixed-methods approach, in which short-term and long-term individual and community-level data were collected from multiple sources through qualitative and quantitative approaches. Our approach was informed by the Kirkpatrick model of evaluating training programs, which emphasizes examining the levels of participant reaction and learning, in addition to impact on behavior and overall performance [20]. The researchers who carried out the evaluation interviews were independent of the staff who coordinated or who delivered the training.

#### Knowledge assessment

At the time of implementation, we measured changes in participants’ knowledge through administering tests before and after the intervention. The pre- and post-tests were administered in person during the training. The tests were developed by two subject matter experts who also developed the content for MUH certificate.

#### Course evaluation

By the end of the training, course evaluation self-reported surveys were administered to participants in-person. This survey aimed to assess participants’ satisfaction with the courses, the instructors, and the certificate as a whole. Participants were also asked about their recommendations to improve the training. The course evaluations included 20 quantitative questions rated on a 5-point Likert scale addressing the evaluation of the course and the instructor, and 3 open-ended questions asking for feedback on the instructor, their course expectations, and recommendations for improvement.

#### Semi-structured interview

We also collected long-term data on the training’s impact through conducting semi-structured (see Table 2) interviews, which lasted between 8 to 15 min, with randomly selected participants from both trained cohorts. We interviewed Syrian (*n* = 4) and Lebanese (*n* = 4) women CHW whose age ranged from 18 until 45. The interviews were conducted in July 2020, included 9 questions, and aimed to assess the experiences of participants with the training, their knowledge and practices in women’s health over the past year, and their experiences fulfilling their roles as CHW to support community members with issues related to women’s health. In keeping with the social distancing measures and mobility restrictions imposed by the COVID-19 pandemic, all interviews were conducted over the phone, recorded following participants' consent, and transcribed verbatim in the original language.

#### Focus group discussion

Finally, we conducted one focus group (see Table 2) that lasted for around 1 and a half hours, with members of the community (*n* = 9), recruited from community centers within the catchment area, who did not enroll in the program, but who were in contact with the trained CHW. Community members were all Syrian and Lebanese females within the age range of 18 to 50. The focus group was conducted in July 2020, included 4 questions, and aimed to assess the community-level impact of the training, almost one year after it was conducted. Since it was challenging to arrange for a virtual focus group session due to the limited resources available for the target groups, our research team conducted the session in-person in a safe and private setting at a local community center in their environment (Table 1).

**Table 1:** Semi-structured interview and focus group guides

Tool	Questions
Semi-structured interview guide	1- Describe your learning experience during your participation in the training
	2- How did the blended learning modality influence your learning process?
	3- Describe your knowledge in Women’s Health after your participation in the training
	4- Describe your practices in Women’s Health after your participation in the training
	5- How did the training impact your capability to learn new skills?
	6- In your opinion, what are the strengths and weaknesses of the training program?
	7- Explain whether or not the certificate on Women’s Health prepared you to respond to your community’s health needs as a community health worker
	8- Since your participation in the Women’s Health training, please describe your experience referring members of your community to receive health services
	9- Describe the extent to which your contributions as a community health worker meet the expectations of your community members in terms of trust and quality of care
Focus group guide	1- Describe your and your community’s experiences in accessing services or information related to women’s health
	2- To what extent do you trust the health services delivered by the community health workers regarding women’s health after the training?
	3- Describe the availability and quality of health services regarding women’s health in your community after the training
	4- To what extent are the trained community health workers responding to the needs of your community with regards to women’s health?

### Analysis

The quantitative data such as the knowledge assessments and course evaluations were entered, managed, and analyzed on the Statistical Package for the Social Science (SPSS). This data was reported in terms of frequencies and percentages, and means were compared through t-tests. As for the interviews and focus group data, they were transcribed verbatim in their original language by the research team and were analyzed on the Excel software. All participants were given codes to protect their anonymity. While the evaluation approach was informed by the Kirkpatrick model [20], our data analysis was informed by qualitative content analysis technique, and followed an inductive approach [21, 22]. After open coding, preliminary codes were developed by two researchers. Codes were then grouped into broader categories based on how they related to each other. Next, themes were created based on emerging patterns from the analysis. The authors met regularly to discuss, analyze, and review the coded responses.

With the exception of one author living in the United States, our team consisted of researchers based in Lebanon who specialize in the fields of medicine, women’s health, public health, global health, psychology, and epidemiology. The research team has extensive experience with capacity building in low-resource and fragile settings, and on working with refugee populations, which positively influences our interpretation of the data given our cultural competence and in-depth understanding of this phenomenon and population.

## Results

**Fig. 1:**
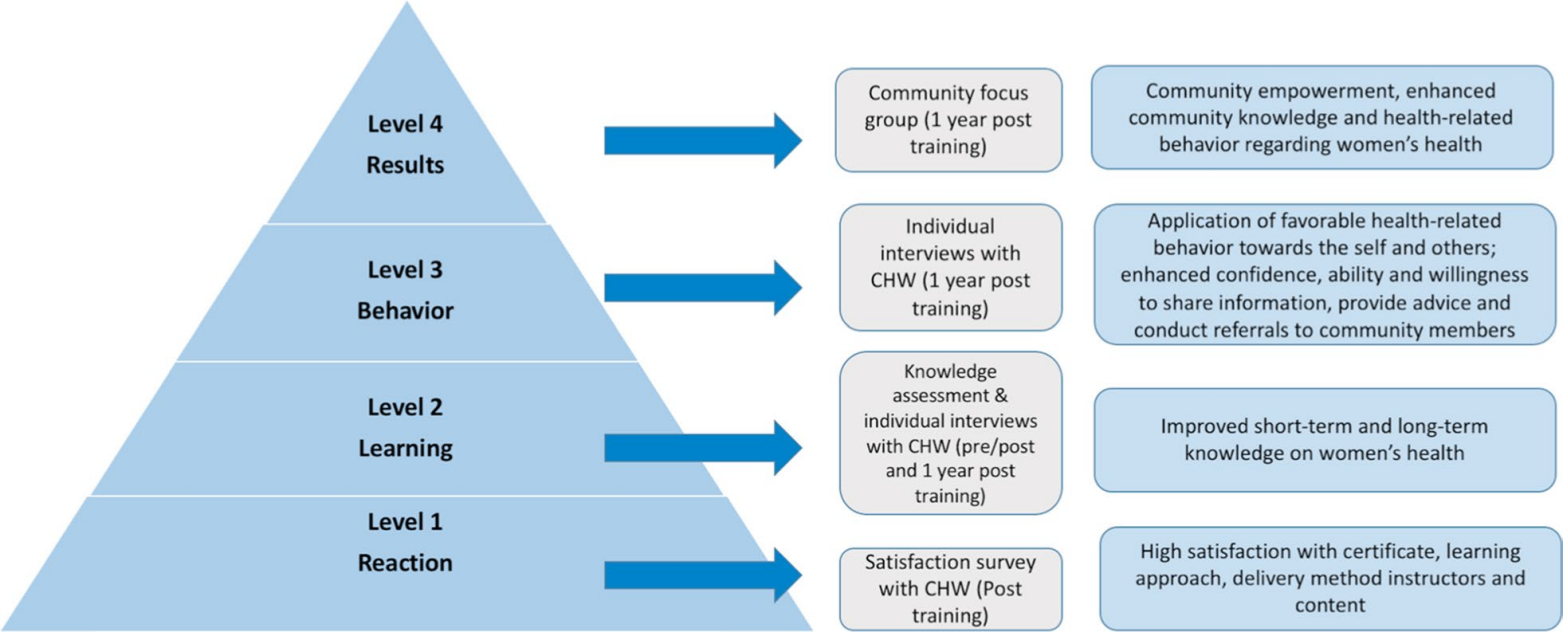
Organization of results through the Kirkpatrick model

**Fig. 2:**
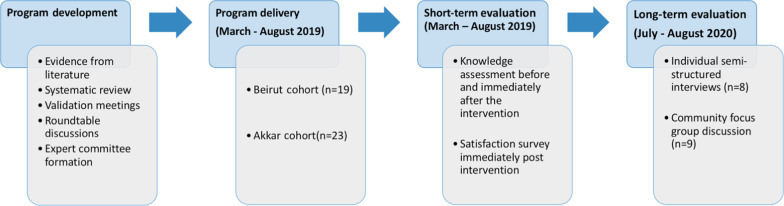
Program implementation and evaluation flowchart

We present the results of the collected data in terms of short-term and long-term outcomes, as informed by the Kirkpatrick model (see Fig. 1). The short-term outcomes include participants’ reactions and learning and were measured by knowledge assessments administered pre and post the courses, along with a course evaluation survey that was administered at the end of the certificate. The long-term outcomes include behavioral changes and results at the community level, were measured by semi-structured interviews and a focus group conducted at the 1-year time period and were triangulated and merged together in the analysis (see Fig. 2). In this section, CHWs refer to participants that attended the training program, whereas community members refer to individuals who participated in the focus group or who are part of the community at large.

### Short-term outcomes

#### Knowledge assessment

Participants were administered an exam prior to and post receiving the training in order to measure the effectiveness of this certificate on their knowledge of the subject matter. In the Beirut cohort, post-test (*M* = 74.71, SD = 8.32) were statistically significant higher that the pre-test scores (*M* = 46.81, SD = 8.76); t(27) = − 13.347, *p* = 0.000, thus, indicating that the knowledge of participants increased after attending the training. In the Akkar cohort, post- scores (*M* = 87.96, SD = 5.51) were statistically significant higher that the pre-test scores (*M* = 51.34, SD = 9.21); t(21) = − 16.714, *p* = 0.000, thus, indicating that the knowledge of participants increased after attending the training.

### Course evaluation survey

With regard to the evaluation of the instructor’s performance, all participants agreed that the instructor treated the students positively and was well-prepared for the course. The majority of the participants also agreed that the instructor was knowledgeable in the course content (96.2%), was able to communicate the course’s information well (92.9%) and was able to stimulate students’ interest in the course (92.5%). In addition to that, most participants reported that the instructor provided a good connection between theory and practice (96.1%), encouraged students to think independently (96.3%), and was able to manage the time well (96.5%). With regard to the teaching effectiveness, all of the participants reported that in general, the instructor’s teaching process was effective (see Table 2).

**Table 2:** Course evaluation data

	Participants evaluation
Instructor feedback	%
Well-prepared for the course	100
Knowledgeable in course content	96.2
Communicated well course’s information	92.9
Provided good connection between theory and practice	96.1
Stimulated interest in the course	92.5
Treated students positively	100
Encouraged students to think independently	96.3
Managed time class well	96.5
Instructor’s general teaching effectiveness	100
Course feedback	
The courses objectives and requirements were clearly presented	100
The set goals of the course were completed	100
The course sequence was logical and appropriate	92.6
The amount of work required for this course was relevant	100
The course exercises and homework were related to the subject	100
The speed of material presentation was appropriate	96.3
I learned from this course something that I perceive as valuable	96.5
The classroom discussions positively contributed to learning this course	100
The course enhanced my interest in the topic it introduced	100
Good Course quality in general	96.2

Answers to open-ended questions	
Feedback on instructors	Positive feedback on course instructors. Instructors possessed expertise in the course content, ability to provide support and encouragement, ability to lead group discussions, and ability to clearly communicate their knowledge
Course expectations	Course met participant’s expectations. Participants acquired new health-related information
Improvement suggestions	Need for more practical approaches throughout the courses. Calls for adding first aid courses, nursing courses, applied medical courses, wider-ranging courses tapping into more diverse health topics beyond women’s health. Expressed interest in having additional time for practical applications of the learnt material

As for the course content, all participants agreed that the course’s objectives and requirements were clearly presented and that the course's goals were met. All of the participants also agreed that the amount of work required for this course and the given exercises and homework were relevant, the classroom discussions positively contributed to the learning process and that the course enhanced their interest in the introduced topics. Moreover, a large number of the participants (92.6%) agreed that the course sequence was logical and appropriate and the speed of the material presentation in the course was allocated appropriately (96.3%). Similarly, the majority of the participants reported that they have learnt from this course something that they perceive as valuable (96.5%), and 96.2% of the participants agreed that the course was of good quality.

### Long-term outcomes

Since this is an evaluation of the impact and effectiveness of the training, we triangulated the results of the semi-structured interviews and focus group, and reported them under two categories, namely strengths and challenges (see Table 3).

**Table 3:** Presentation of qualitative data and emerging themes

Category	Theme	Code
Strengths	1- Access to education	First time experiencing taking a course on women’s health
		Easy access and exposure to new information
		Enhanced opportunities with regard to gender considerations
		Novel insights that clarified misconceptions about women’s health
		Access to information that they otherwise wouldn’t have been able to receive
	2- Learning modality	New experience of receiving information through videos in a mobile classroom environment
		Having pre-recorded sessions mixed with lectures was helpful to deliver information in a simple manner
		Interactions with the TA were most favored
		Participants appreciated the opportunity of seeing doctors conducting actual practice in the community
	3- Knowledge acquisition	Increased knowledge on women’s health that helps with care for the self and for others
		CHW had limited knowledge and low-level education in general. This training helped CHW learn new information on women’s health and offered, in general, new opportunities for education
		Acquisition of new health knowledge following interaction with CHW (through group sharing of personal experiences, informal conversations) who were family members, friends, or acquaintances from the community
	4- Health-related behaviors	Increased ability to help others in the family and in the community regarding women’s health issues by discussing common topics, giving tips, and clarifying misconceptions
		More confidence to share knowledge with friends, family, and the community
		Increased curiosity to learn more by navigating websites and other sources
		Influence on the health practices of family members and community members in touch with CHW
		Because the information was credible since it was delivered by a trusted institution, this gave more confidence in sharing the knowledge and justifying how and why this information is trusted to others
		Change in health-related behavior among community members following interaction with CHW such as increase in screening, increase in referral, increase in self-management, better attention to details related to women’s health such as period, birth control, screening, etc.
	5- Individual and community empowerment	Creation of a stronger sense of community through grouping CHW and community members together in the training
		Important opportunities for self-development and for positive interactions with host community members
		Increased the confidence and strength of CHW because they had something new to offer the community and they had a sense of purpose now
		CHW played an active role in transmitting information to people in their surroundings
		Community members trusted this advice since information came from a trusted and credible source
Challenges	1- Gender considerations	More gender consideration should have been made to have women be TAs as opposed to a man given the sensitive nature of the topic
	2- Learning modality	Need for more engaging and interactive approaches with TAs
		Videos were helpful to deliver information but were not enough to sustain attention
		Need to emphasize on practical and hands-on approaches
		Reduce information and focus on necessary information, increase engagement and discussions
		Need to have shorter trainings so that people can still attend while take care of primary responsibilities such as work and family duties
	3- Long-term retention	There is a need for participants to have easier access to the material they learned after the training to enhance long-term retention
	4- Community reactions	Some members were not receptive and had negative reactions when receiving this kind of information
		Some members were jealous because CHW had an opportunity to learn whereas they did not
		Sometime not being taken seriously or not having credibility when discussing health matters with community members
		Trust regarding the services largely depending on others. Some trusted CHW and wanted to learn, others ridiculed it
		Overall trust in the advice and health information shared by the CHW and establishment of credibility when copies of the learning material and the certification by AUB and GHI were shared
	5- Access to health services	There is generally lack of access to healthcare services due to several issues such as limited availability, limited ability to finance them, having to travel long distance
		Lack of free health services, community members largely struggled to find services (free or cheap ones that they could afford or that had access to given geographical challenges) and they reported lack of security
		Only reported free services were cancer screening in rare times throughout the year
		Despite having new access to health-related information and despite suggestions for referrals to healthcare services, community members felt contradictions

#### Strengths

Based on the results of the semi-structured interviews and focus group, five themes under the strengths category emerged from the qualitative analysis: (1) access to education, (2) learning modality, (3) knowledge acquisition, (4) health-related behaviors, and (5) individual and community empowerment.

### Access to education

Almost all CHW mentioned that this was their first time undergoing such a learning experience. This was, to a large extent, a rare opportunity for them to learn about women’s health topics and to receive such formal education. Importantly, they mentioned that the program, having adopted a mobile delivery method, exposed them to new, relevant, and essential information on women’s health, that they otherwise would have not had the opportunity to receive. This is because they resided in remote and/or low-resource settings in Lebanon, which had very little access to health-related education from formal and credible sources.

*“I.L.A.7: I was encouraged to take this training because women's health is limited here. We don’t have much awareness about this topic, so I wanted to educate myself on this subject and be knowledgeable and of help to others.”*

It also seemed that the training had a ripple effect across community members, and indirectly benefited them also, especially that some of the learning material was shared as physical copies between individuals who did not attend the program. Accordingly, several participants from the focus group expressed interest in joining future training initiatives themselves and described that their communities were in dire need of having more CHW given the positive impact they were causing around them.

*“F.L.A.1: I wish that more training initiatives would be presented for the community here. The individual herself will take in the information and master it, and then implement it on oneself.”*

### Learning modality

CHW commonly reported that the program was well organized and coordinated, exposed them to relevant and important content on women’s health, and had professional instructors who delivered the material in a simple and engaging manner. Importantly, CHW highlighted that the major strengths of the learning modality were the components that included interactions such as group discussions, engaging activities such as the practical elements, and direct conversations with the instructors.

*“I.S.A.1: ... the doctors provided us with the information in a very simplified way and they were very good with us. When we asked them questions, they were willing to answer us until we understood the answer.”*

CHW also appreciated the chance to actively engage in the training and mentioned that this was very helpful for them to learn. The use of pre-recorded videos and related slides helped organize the content of the training and present it in a visually appealing and simple manner, although this was not heavily endorsed. In addition, some of the CHW described having the opportunity to see health providers working in community settings as especially useful.

*“I.L.A.7: Another point of strength is that they used to take us to the clinics. There were not a lot of patients when we used to go there to the clinic; we would only go over one or two patients, but I still believe this is an experience of strength because we attended the clinics and were able to observe the doctors closely.”*

### Knowledge acquisition

All CHW who were interviewed reported increases in their knowledge on women’s health, with the most commonly mentioned topics revolving around pregnancy, childbirth, dysmenorrhea, health maintenance, gender-based violence, care of older individuals, women’s hygiene, cancer, diabetes, genetic vulnerabilities, and early screening. This knowledge was helpful to them on many levels, starting from understanding basic concepts, to discussing and dispelling common misconceptions, and leading up to them knowing how to apply this knowledge for self-care and care of others around them. It is important to note that many participants had limited to no knowledge on this topic before this training, and many had misconceptions around women’s health.

*“I.S.B.2: The instructor corrected a lot of our misinformation regarding pregnancy and childbirth.*”

The training equipped CHW with the knowledge and confidence to spread valuable information on women’s health to their communities. On the community level, these were shared through personal and informal conversations and through casual interactions, whereby the CHW relayed the material they learned, clarified misconceptions, and provided advice to other community members. For example, one community member stated that:

*“F.S.A.2: My sister was enrolled in the training. I was recklessly taking my contraception pills that the doctor prescribed to me following the birth of my two children. After the birth of my daughter, I continued to take my contraception pills without consulting the doctor further. My sister then warned me that this is not the proper way to take the pills and taking it this way can cause uterus cancer. She insisted that I go see a doctor at a specialty clinic to give me the proper instructions for this medication. I didn’t previously know that the medication had to be taken in a specified way; I thought that once the doctor prescribed it to me, I should keep on taking it as is “*

With this acquired information, over the past year some CHW have reported additional curiosity and willingness to learn and improve their knowledge on the topic. Others had maintained some of the material they received from the training in order to help them recall information they may have forgotten over time.

*“I.S.B.3: I also started to associate the information that I learned with additional related information that I searched for on the internet and is related to vitamins and genetic diseases.”*

#### Health-related behaviors

When asked about their health-related practices over the past year, many CHW reported paying attention to details they used to neglect previously such as balancing their diets, having better hygiene, better managing their birth control pills, and being willing to screen for breast cancer more often, among others. These are some of many examples relayed by the CHW with respect to the impact of the knowledge they learned on their self-practices, on and their ability and willingness to pass this on to others. For example, almost all CHW mentioned one or more of their experiences providing advice and sharing health information with family and community members.

*“I.L.B.6: With regard to friends, I am a teacher and I have a lot of teacher friends and each differed from the other with the information they possessed. But when you have information from a credible and reliable source, you feel that you are able to tell them for instance how to take vitamins or calcium. We have an old teacher, so I started to encourage her to take calcium because if she gets a wound or breaks her bone, she will need a lot of time to heal. So, from this perspective, with regard to simple things.”*

Based on their responses, CHW appeared to share health-related information through casual conversations and informal discussions with members of their families and the community surrounding them. In this context, CHW also played an important role in clarifying misconceptions and in recommending increased contact with the health care system for screening, medical check-ups, and related health services.

“*I.S.A.1: I have a friend who has been married for more than two years and a half and still was not able to get pregnant. So, I advised her what she can do and told her that she needs to see a gynecologist to inspect her condition of why she still didn’t get pregnant. And she really did go to the gynecologist and was prescribed with what to do. *“

As a result of these interactions, community members expressed increased motivation to better take care of their own health and of those around them such as their mothers, sisters, and daughters. Access and exposure to new information enhanced the understanding of community members regarding women’s health issues, increased their awareness, and ultimately positively influenced their behaviors. For example, several community members reported an increase in their willingness to screen for breast cancer, and to better self-manage their health.

*“F.S.A.4: Also, with regard to my blood sugar level, I didn’t go frequently to the doctor, once five to six months. Now I gained better knowledge in the proper way of taking the medication, and I started to go to the doctor every month. They prescribed for me a specified medication for sugar level or blood pressure. So it’s better than before.”*

#### Individual and community empowerment

The knowledge acquired from the training helped CHW develop the confidence to engage with others on health issues. It also motivated and empowered them to play an active role in their communities, by taking initiative and being leaders in their environment. The fact that the training was delivered by a credible and highly regarded institution, and by health professionals, gave additional credibility to CHW when interacting with others in the community and reduced skepticism from others. These combined gave CHW a stronger sense of purpose with regard to their role in the community because they were able to help and support others in different ways.

*“I.S.B.3: Also, for example, if a disease topic comes up, I discuss with them what I learnt and tell this, this and that. They take it into consideration knowing that I took the training, and later on they discover that they really did benefit from what I told them.”*

That said, the training helped build stronger relationships between the Syrian and Lebanese individuals and contributed to empowering women in these communities through these interactions and educational activities. Furthermore, one CHW mentioned that the training gave her additional opportunities for education which helped her overcome the limitations her gender posed on her in her community. The training was generally perceived as a strong opportunity for self-development which was highly regarded by the women.

*“I.S.B.2: I liked the training, I really liked it. I felt happy to know something new and something related to me as a woman. The training was personal and served as a means of self-development.”*

### Challenges

Under the challenges category, five themes emerged from the qualitative analysis: (1) gender considerations, (2) learning modality, (3) long-term retention, (4) community reactions, and (5) access to health services.

### Gender considerations

One of the mentioned difficulties was discussing sensitive issues regarding women’s health throughout the training in the presence of an instructor who is a man. Understandably, one CHW requested that more attention should be paid to this issue in future programs to enhance engagement, as she felt that she had to refrain from asking questions and sharing personal experiences because of the associated discomfort. Nevertheless, despite this limitation, the overall feedback on instructors, whether men or women, was positive since both were able to maintain interest and engagement across the training sessions.

*“I.S.B.2: It was a good way that because the woman doctor could not attend, the man doctor came instead. It was hard for us to ask him questions … but we then experienced comfort because the doctors were very supportive when we felt shy to ask questions, but then they provided us with comfort and allowed us to ask the questions comfortably especially that he’s a doctor. This was something really beautiful.”*

### Learning modality

Although the majority of CHW reported positive feedback regarding the modality through which the training was delivered, two important weaknesses were highlighted. The first relates to the use of pre-recorded videos, in that they were perceived as monotonous and, in some instances, not interesting enough to sustain their attention. However, since the instructors were engaging, and habitually initiated group discussions throughout the sessions, this made up for the aforementioned limitation because CHW were able to ask questions and interact with them.

*“I.S.B.3: Honestly, we did like the training, but we wished the instructor that was standing in front of us would have explained to us the lesson rather than letting us watch a video from the laptop or the screen. This instructor’s method at times felt boring, you know.”*

The second weakness according to some CHW was the fact that too much information was being delivered to them, and that the sessions were relatively lengthy. In this regard, CHW recommended that the sessions be shorter to allow learners to attend to other pressing responsibilities such as work or family duties while learning, and for the information to be delivered in a more concise fashion.

*“I.S.A.8: If the length of the training could have been shorter because we have other responsibilities and duties. The training was maybe from 9:00 till 2:00*.”

#### Long-term retention

As apparent during the interviews, many CHW had some trouble remembering much of the information they learned during the training a year ago. Understandably, CHW remembered educational material that they practiced in their daily life, and these were the ones they recounted and gave examples about during the interviews. That said, several CHW highlighted the importance of trainees being supplemented with additional material upon termination of the sessions in future training programs so that they may revisit them when needed.

*“I.S.B.2: My information thank God became better but with regard to knowing the information by heart, I had to go back to the training materials and review them again back and forth.”*

#### Community reactions

Although a major part of their roles as CHW was to engage with other members, relay health-related knowledge, and improve health-related practices in their respective communities, CHW also highlighted important challenges throughout this past year on this community level. Of major importance was the fact that CHW perceived some community members showing negative reactions regarding them for several reasons. For instance, some CHW reported that some members of the community did not trust them, were skeptical towards their shared information, or did not take them seriously when discussing health-related information. One CHW even reported that some members of the community were envious for not having had access to these training sessions themselves.

*“I.S.B.2: ...But some people honestly possessed arrogant characteristics making them feel like they are more knowledgeable; they did not care about what I had to say especially if they possessed a higher level of education, they would not listen to you regardless if the information they possess is right or wrong.”*

In addition to that, findings of the focus group highlighted criticism of community members regarding the fact that many members could not equally reach CHW and the information they had to offer. One member went on to explain how most interactions happened between those who were closest to the CHW and called for implementation of more training initiatives in their community to overcome this problem. Other members also voiced their interest in participating in future training initiatives and suggested for organizers to have longer recruitment phases to allow other people to learn about and enroll in the program.

*“F.L.A.1: The benefit of the training did not reach equally the members of the community. I obtained the information in a way, and I will provide them to others in another way, and of course from the primary source the individual will benefit more. The benefit from the primary source is different from the information that you obtain through second hand sources; information from one person to another changes. So, I wish there will be more trainings for the community whereby the individual will take in the information directly and implement it on oneself.”*

However, despite these negative reactions, some CHW noted that over time others started listening more carefully to them when they witnessed changes in their health behaviors such as being consistent with healthy behaviors and avoiding risky behaviors, which increased their credibility. Also, the fact that the learning material was certified and provided by a credible institution helped establish additional trust. Finally, the fact that community members perceived CHW as being serious about learning and exerted effort to attend the sessions and communicate the material afterwards, facilitated for CHW to establish additional trust and credibility in their communities.

Overall, most community members reported trusting the advice and health information shared by the CHW. This was largely due to the fact that the CHW received a certification by the program, which was developed by a credible institution, and delivered by professional health workers. In addition, being able to read through the material that was given to the CHW added another level of credibility since they could double-check the information shared with them.

*“F.L.A.3: Of course, I trusted them, especially after community health workers showed me the hardcopy material of the training and allowed me to read through it. So, I became assured that the information they acquired is correct.”*

#### Access to health services

Although an important goal of the training was to increase referrals to health services, community members reported critical contradictions that they experienced. That is, despite having increased awareness of, and access to information on women’s health, community members reported very limited ability to access health services due to multiple reasons. For example, limited availability of health services was highlighted by several members as a result of them living in remote areas in Lebanon, and the associated challenges of having to travel for long distances to seek care, which was seldom possible to them. Other reasons were financial challenges, given the scarcity of free or affordable healthcare services, and the lack of specialized services for women’s health. One of the few services reported to be freely accessible was breast cancer screening, and that was only available at specific time-points throughout the year.

*“F.A.S.2: People visit doctors for medical consultation based on their personal expenses, and generally there are no free medical facilities except for the breast cancer screening tests which are offered every six months or one year are hospital X; the hospital usually sends the announcements via WhatsApp. You feel that some health care services are available, and some are not, and not everything you need is easily accessible. You need to search for it to find it.”*

Moreover, one member expressed limited ability to prevent health problems, whereby she mostly accessed services for treatment when she absolutely had to seek medical care. Therefore, in this regard, the CHW played a very important role in spreading essential information on women’s health that could help others identify basic areas of self-care.

## Discussion

Task-shifting has emerged in recent years as an impactful and cost-effective strategy to address health disparities in fragile settings through training non-specialized workforce to deliver basic health education and services to low-resource communities [11, 13, 23]. Many studies have reported the utility of this approach across a range of settings and health outcomes such as for communicable diseases, non-communicable diseases, mental health, among others [12, 24–27]. However, there exist very few studies evaluating the long-term impact of such interventions on women’s health in the Middle East, especially for those that adopt an innovative mobile approach such as MUH. Accordingly, there is a strong need for data on this issue to inform donors, professionals, and policy-makers given the scarcity of these evaluation reports in the literature. In this study, we evaluated the long-term impact of the first MUH certificate on women’s health, on the individual and community levels. We used multiple sources of qualitative and quantitative data, which we triangulated in our analysis, interpretation of the findings, and recommendations.

Overall, this study points towards the women’s health certificate being successful and impactful based on several findings. Most importantly, it enhanced the access of participants in both cohorts to formal education and learning opportunities on the topic of women’s health that they otherwise would not have received. This is because the training program was designed to be innovative in its delivery, being mobile, and therefore easily accessible to women from remote low-resource settings in Lebanon who had very few opportunities for health education. In turn, this exposed them to novel and essential information that is relevant to their daily lives, and which they may utilize to improve their health and that of those around them [13, 28]. Based on our findings, these educational materials obtained by the CHW were often circulated verbally and in their physical forms across community members, whereby this newly acquired information had a ripple effect across the community.

Furthermore, most of the features of the adopted learning modality were reported to be helpful in the delivery of the training. For example, the interactive features, the direct conversations with course instructors, the group discussions, and the practical components were highlighted as major strengths of the training. It is possible that these aspects were conducive for active and experiential learning, since they allowed participants to better engage with, and relate to, the learning material, ask questions, and apply them [29]. Because this was a certificate on women’s health, it was crucial for participants to be able to relate to the content in an engaging manner so that they may better understand how to apply them in their lives and how to share them with others. On the other hand, one important challenge was reported in relation to the pre-recorded videos, since although they facilitated the delivery of health-related education, they were perceived as being monotonous. It is possible that participants felt disengaged with this approach because it lacked a personal element and counteracted their expectations from the training. However, the fact that they were still able to interact with the teaching assistant made up for this minor shortcoming. Potentially, pre-recorded videos could be more effective and engaging if they included an interactive component.

It was clear from the knowledge assessments, interviews, and focus group, that significant knowledge was gained by participants and the community at large. The pre–post tests showed increases in short-term knowledge on women’s health by participants in both cohorts, whereas the interviews highlighted some long-term knowledge retention over the past year; this is consistent with similar studies in the literature on training CHW [30, 31]. Although some participants showed some difficulty remembering some of the content during the interviews, they seemed to mostly retain information that they used in their daily lives to manage their health or to support others around them. For example, basic knowledge and tips that could be applied on topics of pregnancy, childbirth, dysmenorrhea, general health maintenance, gender-based violence, women’s hygiene, cancer screening, among others were commonly mentioned by participants during the interviews. In addition to that, the focus group suggested that most of this information was transferred to community members by the CHW through sharing of personal experiences, direct interactions between both groups, and casual conversations. This is important because the aim of training CHW is to prepare them to spread health-related education and basic services to the wider community [16].

In general, the newly acquired content was pivotal to improving the knowledge and practices of the community in women’s health issues. Reports of improved self-care behaviors, sharing of advice, clearing of misconceptions, and referrals to health care services were commonly mentioned during the interviews and the focus group. Ultimately, this points towards the fact that the MUH program may have been an important intervention for the community at large and was beneficial over time. However, despite this positive impact, members of the community faced significant difficulties accessing health services, and in many instances, applying the knowledge they had gained directly or indirectly from the CHW. According to them, access to health services beyond the tips and advice provided by CHW was limited due to financial and geographical barriers, and due to lack of available specialized services in their areas.

Finally, the MUH program was able to empower the participating women and the community at large since it supported them in developing the confidence to engage with health-related issues and to take care of each other. In fact, community members expressed much interest in wanting to participate in future training programs. The training motivated CHW to play an active role in their communities and gave them a renewed sense of purpose. Although some difficulties were faced in establishing credibility in their communities, most of the CHW were able to gain the trust of others because of the certification they received, their consistency in their behavior, the credibility of the institution that delivered the training, and the fact that they had used physical copies of the learning material when sharing the information. Finally, the training contributed a step forward towards supporting relationships between Syrian and Lebanese individuals, a relationship previously reported to be tense [32], as a result of their engagement and interactions in educational activities.

## Conclusion and Limitations

Despite many strengths of our study, such as adopting a long-term evaluation approach for the intervention on the individual and community levels, using multiple sources of data, and using an established evaluation framework, some limitations should be acknowledged. For example, we did not collect any quantitative data on health outcomes or other health-related indicators such as service use before and after the intervention to be able to comprehensively understand its wider implications. We mainly relied on qualitative methods to assess the impact of the program, however future research may be essential to examine population-level impact using quantitative means to better understand the wider implications of similar training programs. In addition to that, it is worth mentioning that participants might have experienced biases such as social desirability when completing the interviews or focus group discussions because they tend to receive services such as educational opportunities from the team at the GHI; to minimize this, participants were assured that their participation, or lack thereof, would not impact their relationship with the team.

More evaluation reports of task-shifting in low-resource settings are needed to better understand the impact of such interventions, the extent to which they may positively influence health education, behaviors, and outcomes in these communities, and how to optimize their design, delivery, and evaluation. Our team is currently implementing the full MUH program, delivering all three certificates on mental health, non-communicable diseases, and women’s health to displaced populations in different low-resource settings in Lebanon.

In conclusion, both the trained CHW and members of the community at large endorsed this training as being effective and as having a major positive impact on the community one year after it was implemented. It was evident based on our data that this training addressed an important gap and delivered valuable information to low-resource settings that would have otherwise not received such learning opportunities. Our findings funnel into 4 major policy and programmatic recommendations for improvement of this and other programs implemented in contexts with similar cultural and development landscapes:

- First, and on a policy and macro-level, it is crucial to provide more funding to deliver more training programs on a variety of health topics to be able to reach more individuals and prepare them to be community health workers in their settings. In this regard, and to ensure that more persons have an opportunity to become community health workers, we recommend longer recruitment phases, and actively promoting their role in all areas to be covered.
- Second, more emphasis should be placed on interactive components, group discussions, and practical approaches in order to maximize engagement and optimize the learning experience. In addition, it may be helpful to reduce the length of the sessions and reduce the delivery of non-essential educational material so that the focus remains on key health messages. That said, it is very important to deliver concise material that has direct applications in participants daily life, and that could be useful for care of self and of other people.
- Third, during and after the delivery of the training, participants may benefit from having comprehensive summaries of the learning content available to them in soft and hard copies. This is essential for long-term retention of knowledge, to help them share the knowledge with others and circulate it within their communities, and to help them enhance their credibility when discussing with others who may show skepticism.
- Fourth, it is important to provide participants resources for referrals so that they may use them in their roles as community health workers when needed. These resources could potentially list affordable or free available services, in addition to specialized services in their catchment areas.

## Data Availability

The data used in this study are available from the corresponding author on reasonable request.
